# Manual of Physiology

**Published:** 1857-09

**Authors:** 


### BOOK NOTICES.

*Manual of Physiology. By William Senhouse Kirkes, M. D., Fellow of the Royal College of Physicians, etc., etc. A new and Revised American from the last London Edition, with two hundred Illustrations. Blanchard and Lea, Philadelphia*, 1857.

This is a new edition of a work already pretty well known to the profession. It is neatly printed and substantially bound, containing 584 pages, and very well illustrated with wood cuts. Without any of the fulness and completeness presented by the works of Carpenter, and Todd, and Bowman, this is, nevertheless, a well digested and useful manual, which will be found very convenient both by the student and practitioner.

